# Morpho-Physiological Adaptation of Sunflower Hybrids to Varying Plant Densities

**DOI:** 10.3390/plants14223446

**Published:** 2025-11-11

**Authors:** Antonela Markulj Kulundžić, Ivica Liović, Aleksandra Sudarić, Tomislav Duvnjak, Maja Matoša Kočar, Ivana Varga, Anto Mijić

**Affiliations:** 1 Department of Industrial Plants Breeding and Genetics, Agricultural Institute Osijek, 31000 Osijek, Croatia; ivica.liovic@poljinos.hr (I.L.); aleksandra.sudaric@poljinos.hr (A.S.); tomislav.duvnjak@poljinos.hr (T.D.); maja.matosa@poljinos.hr (M.M.K.); 2 Faculty of Agrobiotechnical Sciences Osijek, J.J. Strossmayer University of Osijek, 31000 Osijek, Croatia; ivana.varga@fazos.hr

**Keywords:** *Helianthus annuus*, agrotechnical measure, JIP-test, photosynthesis, biomass, yield, oil, correlation

## Abstract

This study evaluated the responses of five sunflower (*Helianthus annuus* L.) hybrids (Surimi CL, Integral CL, Alexa SU, Neta SU, and Davero SU) to three planting densities (84,034, 68,027, and 57,143 plants/ha) in terms of agronomic performance and photosynthetic efficiency. Higher plant density reduced leaf area and seed weight but enhanced uniformity of head formation. Among the tested hybrids, Integral CL and Surimi CL demonstrated superior performance under high density, maintaining higher chlorophyll content, photosynthetic activity, and yield stability. In contrast, Davero SU performed best under low density, characterized by greater leaf expansion, seed filling, and overall productivity. These findings highlight the potential of integrating physiological and agronomic traits to inform hybrid-specific planting density optimization under diverse environmental conditions.

## 1. Introduction

Sunflower (*Helianthus annuus* L.), which belongs to the *Asteraceae* family, ranks among the leading global oilseed crops, alongside palm, soybean, and rapeseed. Its significance is evident from the extensive cultivation area, which in 2023 covered approximately 30 million hectares worldwide, resulting in an estimated production of 58 million tons. Within the same year, the European Union made a notable contribution, cultivating around 4.7 million hectares and producing around 10 million tons of sunflower seeds [1]. Over the past decade, global sunflower production has increased by approximately 50%, while in the European Union, it has grown by 30%. Sunflower oil is considered one of the highest-quality vegetable oils, valued for its excellent nutritional and energy properties [2]. Consequently, the demand for its production continues to rise.

One of the key reasons for the expansion of sunflower cultivation is its exceptional tolerance to drought conditions, both in soil and air, compared to other spring field crops. This trait is becoming increasingly important as climate change intensifies drought occurrences globally [3,4]. Sunflower is a heliophilous plant that, according to various classifications, is classified as a subtropical or even tropical cultivated plant. It is characterized by a strong, well-branched root system with high water absorption capacity, as well as a specific anatomical structure of the stem and leaves [5]. However, despite these adaptations, continuous research on sunflowers remains essential because changes in climate and weather conditions in a short period are extremely large, which is certainly reflected in their growth and productivity. Such shifts create new environmental conditions, altering the intensity of stress factors on sunflower plants and reshaping genotype-environment interactions [6]. For this reason, the present study included five commercially relevant sunflower hybrids representing both Clearfield (imidazolinone-tolerant) and tribenuron-methyl-tolerant technologies. These hybrids differ in maturity group, stress resilience, and overall agronomic performance, enabling the assessment of genotype-specific responses to variable environmental conditions.

Each environmental factor, whether it be agrotechnical measures, weather conditions, or soil properties, individually or synergistically influences the expression of traits in every sunflower genotype [7,8,9,10]. Agrotechnical measures encompass a range of mechanical, physical, chemical, and biological interventions applied to agricultural soil. Besides preserving soil fertility, these measures are designed to tailor existing agroecological conditions to meet the biological requirements of the crop, thereby maximizing its genetic potential. Agrotechnical measures further aim to promote the optimal growth and development of plants while ensuring that the yields justify the quantity and quality of the labour and resources invested [11]. Researchers are continually exploring ways to assist plants in adapting to adverse conditions through agrotechnical strategies, thereby minimizing the negative impacts to the greatest extent possible [12].

Sowing is a crucial agrotechnical measure that not only defines the vegetative area of cultivated plants but also ensures the optimal absorption of essential resources, such as nutrients, water, and light, from both soil and air. The sowing design should account for the genotype-specific requirements of the sunflower hybrids as well as the local agroecological conditions to achieve the desired agronomic performance. In high-density plantings, competition among plants intensifies, causing changes in the morphology and physiology of plants [13]. These changes include modifications in leaf area, plant height, head diameter, and stem diameter. Furthermore, sunflower exhibits both phenotypic and photosynthetic plasticity, allowing it to adjust its growth, resource allocation, and photosynthetic performance in response to varying environmental conditions. Photosynthetic plasticity, in particular, refers to the ability of a plant to adjust to modulate light capture, energy conversion efficiency, and electron transport in response to factors such as light availability, water status, and planting density. This plasticity enables certain hybrids to maintain relatively stable photosynthesis and optimize resource use under stress or crowding conditions.

A dense canopy with a high leaf area index allows for shading of the soil surface, reducing evaporation and maintaining lower soil temperatures [14]. Defining the optimal plant density is therefore critical, as it affects not only composition and resource utilization but also the overall architecture of the plant, seed yield and other agronomic properties. Factors such as temperature, soil fertility, moisture availability, and genotype play key roles in defining optimal density, and achieving the ideal plant composition can maximize grain yield and stability, particularly under the challenges posed by climate change [15,16,17,18,19,20].

The adoption of rapid and non-destructive methods, such as chlorophyll *a* fluorescence (ChlF), allows for detailed assessment of photosystem II functionality and photosynthetic efficiency under both controlled and field conditions [21]. ChlF measurements are widely applied to investigate plant responses to various environmental stresses, such as extreme temperatures, inadequate or excessive light, salinity, drought, herbicides and heavy metal contamination [22,23,24,25,26]. Studies have shown that agrotechnical practices, including row spacing and plant density, significantly influence photosynthetic performance [27]. Odho et al. [28] highlight that, unlike some other crops, sunflower has no lateral branches or shoots that can fill the spaces between plants, making optimal row spacing crucial for maximizing photosynthetic efficiency.

Changing environmental factors and agrotechnical practices not only influence the photosynthetic efficiency of crops but also affect key yield components. For example, reducing a planet’s density increases the number of branches, which is considered the compensatory primary mechanism for a smaller number of plants. However, although this branching can lead to more pods and seeds per plant, the overall yield is often still lower compared to denser plantings [29]. In contrast to this study, Ferreira et al. [30] have shown that increasing plant density tends to decrease the number of pods, seeds, and even the seed weight per plant. These variations in yield components are closely linked to the amount of light available, which directly influences the development of these traits [31]. Thus, understanding the interplay between environmental conditions, plant density, and physiological responses, such as those measured by ChlF, provides a comprehensive picture of how to optimize both photosynthetic performance and crop yield under varying agroecological conditions.

Available research focuses on general responses in yield and morphological adaptations of sunflowers to different plant densities, and limited information is available on monitoring the physiological mechanisms underlying hybrid-specific photosynthetic performance. There is a lack of research examining the photosynthetic responses of different sunflower hybrids at key growth stages and their relationships with agronomic traits. This information is essential for identifying hybrids with superior adaptability and optimizing planting density strategies under changing environmental conditions. Such information will advance the steps of the long-term sunflower breeding process.

The primary intention of the research was to evaluate the morphophysiological and agronomic responses of sunflower hybrids to different plant densities to identify optimal conditions for maximizing productivity. Specifically, the study aimed to: (a) assess how varying plant densities affect photosynthetic activity and yield components of sunflower hybrids; (b) determine correlations between physiological parameters and agro-economic performance under contrasting densities; and (c) identify hybrid-specific adaptive responses that indicate tolerance or sensitivity to crowding stress. We hypothesized that sunflower hybrids differ in their morphophysiological plasticity and that optimal plant density depends on hybrid-specific traits that influence photosynthetic efficiency and yield stability.

## 2. Results and Discussion

### 2.1. Agronomic Properties

Analysis of variance (ANOVA) for the source of variability treatment revealed a significant difference for all agronomic properties except for the number of leaves and hectoliter mass (Table 1). Hybrid as a source of variability was not significant only for head diameter. The interaction of treatment and hybrid revealed significance only for grain yield, oil content, and oil yield properties.

**Figure 1:**
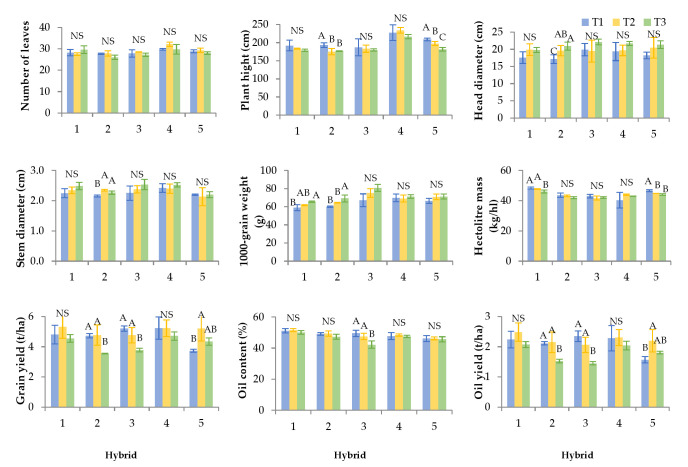
Agronomic properties per hybrids (1—Surimi CL, 2—Integral CL, 3—Alexa SU, 4—Neta SU, 5—Davero SU) in different plant densities (T1—84,034 plants/ha, T2—68,027 plants/ha, and T3—57,143 plants/ha). Error bars represent standard deviations. The letters above the columns indicate a statistical difference at the 5% level for each hybrid individually between treatments; NS—not significant.

The effect of plant density on the tested agronomic properties is shown in Figure 1. Post hoc Fisher’s test of significance showed that plant densities did not affect the number of leaves for any hybrid. Except for the hybrid Neta SU, the tallest plants were generally observed in the highest planting density (T1).

Plant height differed significantly by plant densities for Integral CL and Davero SU. Integral CL showed an increase in height under the densest sowing (T1), while similar but slightly lower values were recorded at other plant densities (T2 and T3). Also, Davero SU had the highest plant height in the densest sowing (T1), with a tendency for height to decrease in the sparser sowings (T2 and T3).

Head diameter was generally decreased with increasing density, being smallest at the highest plant density (T1) for all hybrids except for Alexa SU. Similarly, stem diameter was lowest in T1 for most hybrids, with exceptions observed in Neta SU and Davero SU hybrids. Significant differences in head and stem diameters were observed only in the hybrid Integral CL, where the smallest dimensions were recorded at the highest density (T1). Significantly larger values were found at both T2 and T3, which did not differ from each other.

The 1000-grain weight significantly differed by plant density for the Surimi CL and Integral CL hybrids. In both hybrids, the lowest 1000-grain weight was observed under the densest planting (T1). Surimi CL showed an increased 1000-grain weight in sparser plantings, while the highest value for Integral CL was recorded at the sparsest density (T3).

Hectoliter mass generally decreased at the highest density (T1). Significant differences were observed in Surimi CL, where higher values were found at T1 and T2, and in Davero SU, which had the highest hectoliter mass in T1 and declining values at lower densities.

Grain and oil yields followed similar trends across hybrids in response to plant density. No significant density effects were observed for Surimi CL and Neta SU. In contrast, denser plantings (T1 and T2) increased grain and oil yields for Integral CL and Alexa SU, while Davero SU responded oppositely, achieving higher yields under sparser densities (T2 and T3).

Oil content was significantly affected by plant density only in the hybrid Alexa SU, which had higher oil content under denser conditions (T1 and T2) and lower values in sparse planting (T3).

These findings are consistent with Odho et al. [28], who demonstrate that denser planting resulted in taller plants, while sparser planting resulted in larger stem and head diameters, higher grain yield per head, and higher 1000-grain weight. This is consistent with the results of this study. However, different results were found for grain yield and oil content. Similarly, Demir [32] reported that increasing planting density increases plant height but reduces stem and head diameters, number of grains per head, 1000-grain weight, and grain and oil yields per plant. In contrast, Yousif and Mohamedzein [33] found that increased density led to a higher number of filled grains, more grains per head, higher grain yield per head, and greater total grain yield. Maklad [34] also noted that sparser planting resulted in greater grain yield per head, more leaves per plant, larger leaf area, thicker stems and heads, and higher 1000-grain weight, while denser planting produced taller plants and greater oil yield. On the other hand, Beg et al. [35] found no significant relationship between plant density and traits such as plant height, head and stem diameter, flowering and grain-filling duration, 1000-grain weight, and grain yield. Marin and Ion [36] emphasized the importance of agroecological conditions in determining optimal plant density. According to their study, under favourable conditions with an adequate water supply, the highest sunflower grain yields were obtained at lower densities. However, under less favourable conditions, denser plantings ensured better growth and higher yields. The results of this study indicate that increasing plant density increases stem elongation but reduces head and stem diameter, 1000-grain weight, and hectoliter mass. On the other hand, it is evident that grain and oil yield responses strongly depend on hybrid characteristics. The study provides new insights into genotype-specific adaptation strategies. It identifies useful indicators of hybrid adaptability, which include head and stem diameter stability and 1000-grain weight plasticity under competitive conditions. Overall, higher plant density results in morphological trade-offs between vegetative and reproductive growth, with the degree of adaptation varying among hybrids. Integral CL and Alexa SU performed best at higher densities (T1, T2), while Davero SU gave better yield at lower densities (T2, T3). The most reliable agronomic indicators were plant height response, head diameter stability, 1000-grain weight and hectoliter mass consistency. These findings highlight that optimizing planting density to match hybrid-specific growth dynamics can increase sunflower productivity and direct breeding toward improved density tolerance and adaptability.

### 2.2. Physiological Properties

ANOVA for the source of variability stage of development revealed a significant difference for all ChlF parameters except for ET_0_/RC. Treatment as a source of variability was insignificant for ET_0_/RC, RE_0_/RC, RE_0_/ET_0_ and (RE_0_ − ET_0_)/[1 − (RE_0_ − ET_0_)]. The hybrid was significant for all investigated physiological properties. The interaction of stage and treatment (S × T), stage and hybrid (S × H), treatment and hybrid (T × H), and stage, treatment and hybrid (S × T × H) showed different significance/insignificance according to the properties that can be seen in Table 2.

In this study, the JIP test was used to track changes in the light-dependent reactions of photosynthesis through three developmental stages of sunflowers (budding, flowering and grain-filling) in the field experiment, where five sunflower hybrids were exposed to growth at different plant densities (T1—84,034 plants/ha, T2—68,027 plants/ha, and T3—57,143 plants/ha).

**Figure 2:**
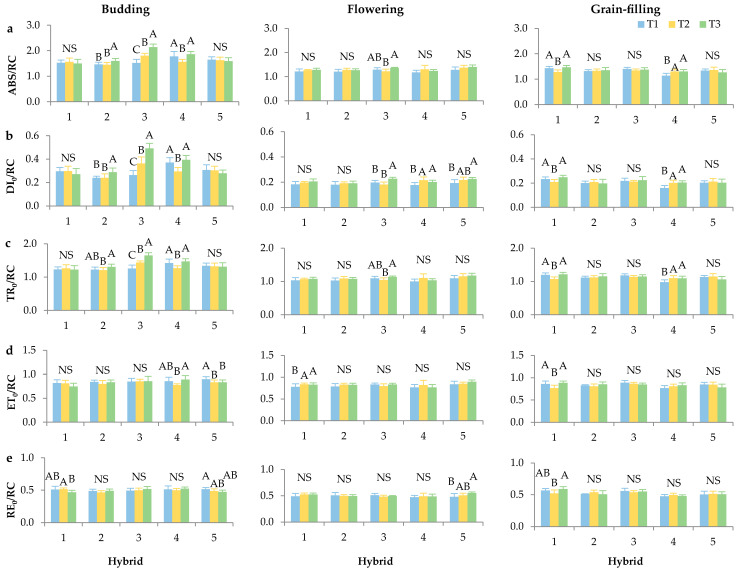
Chlorophyll *a* fluorescence parameters: (**a**) ABS/RC—absorption flux active per reactive centre (RC), (**b**) DI_0_/RC—dissipated energy flux per active RC, (**c**) TR_0_/RC—trapping flux per active RC, (**d**) ET_0_/RC—electron transport flux per active RC and (**e**) RE_0_/RC—electron flux reducing end electron acceptors PSI acceptor side per RC (relative units). Measurements were taken at budding, flowering and grain-filling stages of sunflower hybrids (1—Surimi CL, 2—Integral CL, 3—Alexa SU, 4—Neta SU, 5—Davero SU) grown under different plant densities (T1—84,034 plants/ha, T2—68,027 plants/ha, and T3—57,143 plants/ha). Error bars represent standard deviations. The letters above the columns indicate a statistical difference at the 5% level for each hybrid individually between treatments; NS—not significant.

When observing the parameters ABS/RC, DI_0_/RC, TR_0_/RC, ET_0_/RC and RE_0_/RC across developmental stages of sunflowers, the hybrids exhibited differing responses to the various plant densities, which indicate that the developmental stage strongly determines the values of photochemical parameters normalized to RC of PSII (Figure 2a–e). The budding stage presented higher RC parameter values compared to the flowering and grain-filling stages, with the highest values recorded for ABS, DI_0_, and TR_0_ per RC. The same results were previously determined in research by Markulj Kulundžić et al. [32] on sunflower hybrids. These results suggest an enhanced capacity for light absorption and energy trapping during this critical period of leaf formation and early inflorescence development. Such high PSII efficiency may be interpreted as an adaptive response in which plants at the budding stage maximize light-harvesting for photosynthetic processes and leaf growth, and then shift during flowering and grain-filling toward increased regulation of excess-energy dissipation as resources are reallocated to reproductive organs. This was also established by Huang et al. [37] in their study of the effect of different cadmium stresses on rice.

Moreover, all hybrids showed a similar trend for ABS, DI_0_ and TR_0_ per RC in the budding stage. The hybrid Alexa SU showed the most significant statistical differences, indicating the highest sensitivity to plant densities. Specifically, for Alexa SU, the values of ABS, DI_0_, and TR_0_ per RC increased when shifting from dense to sparse sowing, suggesting that under lower competition for light and nutrients, each plant unit either activates more RCs or redistributes energy flux within PSII. Contrary to these results, Chen et al. [38] found an increase in ABS/RC with increasing maize planting density, suggesting that high planting density inactivated PSII RC and reduced the number of active PSII RC. In contrast, for the hybrids Surimi CL and Davero SU, plant densities did not affect these parameters during budding. Contrary to the described parameters, ET_0_ and RE_0_ per RC did not show any regularities in behaviour depending on the sowing conditions, implying that downstream electron–transport phases and subsequent reduction reactions in PSI are influenced by other factors that are not directly linked to planting density.

In the flowering stage, plant densities did not affect any parameter per RC for the hybrid Integral CL, which indicates its stable photochemical balance and the ability to maintain constant photosynthetic activity despite competition. Only ABS/RC and TR_0_/RC were statistically significant for Alexa SU, with the highest values observed under rare plant density conditions. In contrast, ET_0_/RC was statistically significant only for Surimi CL, while RE_0_/RC was significant only for Davero SU, with increasing values going from denser to sparser sowing.

During the grain-filling stage, Surimi CL emerged as the only hybrid that demonstrated sensitivity, i.e., the greatest photosynthetic plasticity, to plant densities on all parameters per RC. All RC parameters decreased at the optimal planting density (T2). This suggests that in the late developmental stage, this hybrid maximally employs adaptive mechanisms to balance PSII photochemistry with sink strength from the developing grain. In addition, Neta SU showed significant increases in ABS, DI_0_, and TR_0_ by RC, indicating that absorption and energy-trapping phases remain key control points for photosynthesis and late developmental adjustment. Begović et al. [27] examined the responses of winter barley genotypes to environmental variations during the growing season and reported genotype-specific differences depending on the developmental stage. Similarly, the results of the present study revealed that some genotypes exhibited significant changes in several parameters during anthesis, while others showed pronounced responses in both anthesis and the early grain-filling stage. These findings suggest that genotypes exhibit varying sensitivities and adaptive strategies, depending on the phenological stage and prevailing environmental conditions.

**Figure 3:**
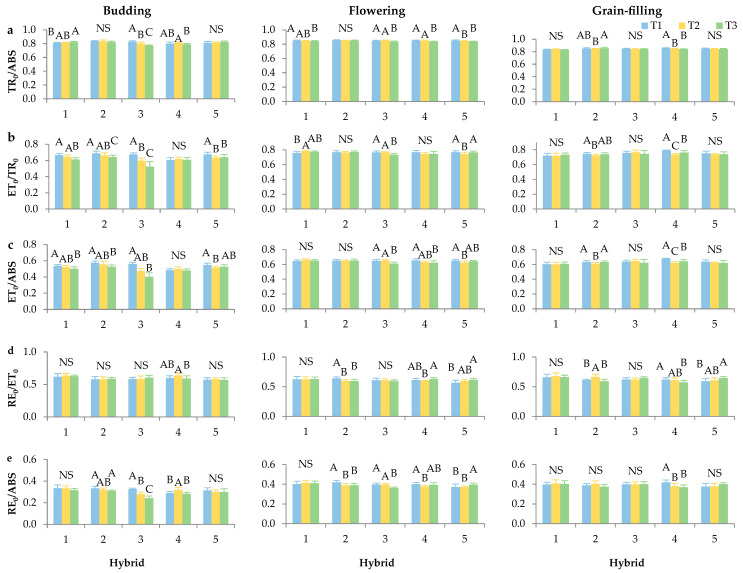
Chlorophyll *a* fluorescence parameters: (**a**) TR_0_/ABS—maximal photochemical quantum yield, (**b**) ET_0_/TR_0_—the probability that a trapped exciton moves an electron into the electron transport chain beyond Q_A_^–^, (**c**) ET_0_/ABS—quantum yield for electron transport, (**d**) RE_0_/ET_0_—the probability that an electron is transported from reduced PQ to the electron acceptor side of PSI, (**e**) RE_0_/ABS—quantum yield of electron transport from Q_A_^−^ to the PSI end electron acceptors (relative units). Measurements were taken at the budding, flowering and grain-filling stage of sunflower hybrids (1—Surimi CL, 2—Integral CL, 3—Alexa SU, 4—Neta SU, 5—Davero SU) grown under different plant densities (T1—84,034 plants/ha, T2—68,027 plants/ha, and T3—57,143 plants/ha). Error bars represent standard deviations. The letters above the columns indicate a statistical difference at the 5% level for each hybrid individually between treatments, NS—not significant.

Similar trends were observed for the parameters TR_0_/ABS, ET_0_/TR_0_, ET_0_/ABS, RE_0_/ET_0_, and RE_0_/ABS, which were examined according to the budding, flowering, and grain-filling developmental stages of sunflower (Figure 3a–e). During the flowering stage, the parameter ET_0_/TR_0_ exhibited generally higher values across all hybrids and plant densities, indicating enhanced efficiency in the transfer of excitation energy from trapped photons to the electron transport chain. An increase in ET_0_/TR_0_ values per growth stage, from booting to anthesis, was also found in barley [27]. In contrast, ET_0_/ABS and RE_0_/ABS showed reduced activity during the budding stage, which is consistent with the research of Begović et al. [27], suggesting limited photochemical efficiency and electron flow at this early stage, which, according to Brestič and Živčák [39], is likely due to the immaturity of the photosynthetic apparatus.

Notably, during the budding stage, hybrids Integral CL and Davero SU showed no significant response in the TR_0_/ABS parameter to varying plant densities. This suggests greater photosynthetic stability or tolerance to planting density at early stages in these genotypes. In a similar context, Mgolozeli et al. [40] on jute mallow, examining the interactive effects of planting density and water availability on the growth, development, total biomass yield, and quality, also reported no significant variation in TR_0_/ABS across different planting densities. Furthermore, ET_0_/TR_0_ and ET_0_/ABS exhibited consistent behaviour across hybrids and densities in this study, indicating that early vegetative stages may buffer against environmental variation. In contrast, RE_0_–ET_0_ was significantly affected only in hybrid Neta SU, implying genotype-specific sensitivity in the efficiency of electron transport to PSI end acceptors.

During the grain-filling stage, the hybrid Integral CL exhibited sensitivity to plant density, as indicated by variations in TR_0_/ABS, contrasting with its earlier stability. This shift suggests a delayed or density-induced stress response that becomes apparent only at later stages, which may be the result of an imbalance between source and sink under higher plant competition caused by seeding density. On the other hand, the hybrids Surimi CL and Alexa SU remained essentially unaffected across all parameters and densities, indicating a robust photosynthetic performance and potential suitability for planting sunflowers in denser sowing. The differential response observed in Neta SU further supports the notion that genotype-specific physiological plasticity plays a critical role in adaptation to agronomic practices.

**Figure 4:**
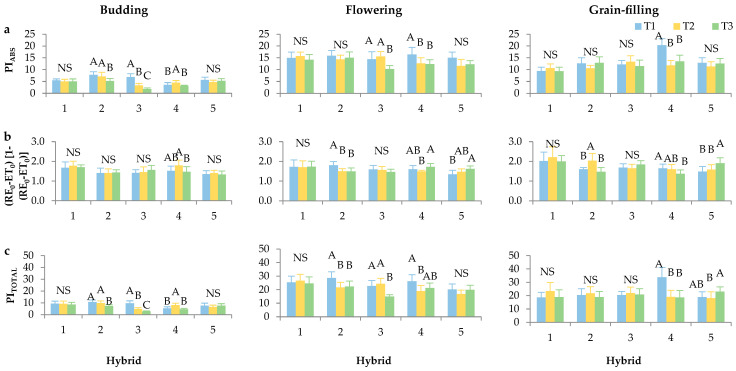
Chlorophyll *a* fluorescence parameters: (**a**) PI_ABS_—performance index on absorption basis, (**b**) (RE_0_ − ET_0_)/[1 − (RE_0_ − ET_0_)]—electron transport from PQH_2_ to final PSI acceptors and (**c**) PI_TOTAL_—performance index for energy conservation from exciton to the reduction in PSI terminal acceptors per budding, flowering and grain-filing stage of sunflower development and hybrids (1—Surimi CL, 2—Integral CL, 3—Alexa SU, 4—Neta SU, 5—Davero SU) in different plant densities (T1—84,034 plants/ha, T2—68,027 plants/ha, and T3—57,143 plants/ha). Error bars represent standard deviations. The letters above the columns indicate a statistical difference at the 5% level for each hybrid individually between treatments, NS—not significant.

The performance index parameters PI_ABS_ and PI_TOTAL_, which integrate multiple aspects of PSII functionality and overall photosynthetic performance [21], exhibited markedly lower values during the budding stage compared to the flowering and grain-filling stages (Figure 4a,c). These findings are consistent with previous research on sunflower, where increases in PI_ABS_ and PI_TOTAL_ were observed at later stages [41,42] and wheat [43].

Furthermore, a consistent pattern of statistical significance across developmental stages and plant densities for most hybrids suggests that both growth stage and plant density substantially influence photosynthetic efficiency. Notably, a decrease in PI_ABS_ and PI_TOTAL_ was observed with increasing plant density. Studies in barley and maize have also documented a consistent decline in PI_ABS_ with increasing planting density, indicating that higher density reduces photosynthetic efficiency, likely due to a dry year [44].

In the budding stage, the Surimi CL and Davero SU hybrids exhibited no statistically significant differences for PI_ABS_, (RE_0_ − ET_0_)/[1 − (RE_0_ − ET_0_)], and PI_TOTAL_ across different plant densities. Notably, (RE_0_-ET_0_)/[1 − (RE_0_ − ET_0_)] was statistically significant only for the Neta SU hybrid.

During the grain-filling stage, Surimi CL and Alexa SU maintained stable performance across all three parameters regardless of plant density, highlighting their potential adaptability and photosynthetic robustness in later growth stages. In contrast, Neta SU again stood out by exhibiting significant differences across PI_ABS_, (RE_0_ − ET_0_)/[1 − (RE_0_ − ET_0_)], and PI_TOTAL_, confirming its higher responsiveness to plant density across developmental stages. These results suggest genotype-dependent strategies for coping with intra-specific competition and may inform hybrid selection for optimal performance under varied sowing conditions.

The greater sensitivity of Alexa SU and Neta SU to planting density likely reflects genotypic differences in plant architecture, leaf anatomy, and resource allocation strategies. Alexa SU invests more in vertical growth, producing taller plants with smaller heads and stems. This limits the partitioning of assimilates towards grain filling, resulting in less stable PSII regulation. In contrast, Surimi CL and Integral CL show more balanced growth and stable photosynthetic performance, suggesting more efficient light capture and gene regulation that supports resistance under competitive conditions.

The physiological traits of sunflower hybrids were strongly influenced by plant density and sunflower developmental stage. In all three tested stages (budding, flowering and grain filling), differences were observed among hybrids in most ChlF parameters. This highlights genotype-specific responses to density. The JIP-test revealed that higher seeding density often reduced photosynthetic efficiency. However, some hybrids maintained stable photochemistry across all planting densities, suggesting distinct adaptive strategies. Integral CL and Surimi CL maintained stable PSII efficiency throughout development, while Alexa SU and Neta SU were more sensitive to developmental changes. Budding was crucial for light capture, while flowering and grain filling reflected hybrid-specific adaptations to competition. The most reliable indicators of adaptability were the parameters ABS/RC, TR_0_/ABS, PI_ABS_ and PI_TOTAL_. These findings suggest that selecting hybrids with stable photochemistry can optimize photosynthetic performance and yield under different densities, providing practical guidelines for breeding and cultivation.

### 2.3. Correlations Between Morphological and Physiological Properties

To understand the relationship between morphological properties and photosynthetic parameters throughout the developmental stages of sunflower (budding—Bud, flowering—Flow, and grain filling—Gf) within different plant densities, correlations were performed (Table S2). The obtained results reveal a complex relationship between morphological growth indicators and physiological processes of the photosynthetic apparatus.

The number of leaves and plant height show a strong positive correlation (r = 0.80), which indicates a related vegetative development of sunflower plants. The interrelationship between plant height and number of leaves observed in this study is in agreement with the findings of Radić et al. [45] and Jing et al. [46], who reported a similar pattern in sunflower and alfalfa. Their results demonstrated that increased plant height led to a greater number of leaves per plant, improved the leaf-to-stem ratio, and influenced key leaf traits, ultimately enhancing the plant’s photosynthetic capacity. However, negative correlations of plant height with parameters of photosynthetic efficiency in the grain-filling stage (ABS/RC r = −0.53; DI_0_/RC r = −0.54; RE_0_/RC r = −0.66; (RE_0_ − ET_0_)/[1 − (RE_0_ − ET_0_)] r = −0.52) suggest a physiological compromise between traits. The observed correlations indicate trade-offs between structural growth and photosynthetic efficiency. Thicker stems were negatively associated with TR_0_/ABS, ET_0_/ABS, and PI_ABS_, suggesting that assimilates invested in stem reinforcement enhance stability but limit PSII efficiency, consistent with reports in maize [47].

The strong positive correlation between head diameter and 1000-grain weight (r = 0.75) confirms that larger heads contribute to higher yield. In contrast, the moderate negative correlation between head diameter and oil content (r = −0.56) suggests a potential trade-off between morphological yield components and the biochemical composition of the grain, which is also reported by Machikowa and Saetang [48]. On the other hand, head diameter is significantly correlated with ABS/RC, DI_0_/RC, and TR_0_/RC parameters, and exhibits negative correlations with TR_0_/ABS, ET_0_/TR_0_, ET_0_/ABS, RE_0_/ABS, PI_ABS_, and PI_TOTAL_ at both budding and flowering stages. These findings suggest that although larger head structures enhance grain filling, they may be associated with a decline in certain aspects of photosynthetic efficiency.

Stem diameter showed statistically significant correlations with photosynthetic efficiency parameters exclusively in the budding stage, indicating that this early developmental stage is critical for structural traits to influence photosynthetic performance. Namely, stem diameter positively correlated with ABS/RC (r = 0.57), DI_0_/RC (r = 0.63) and TR_0_/RC (r = 0.53), indicating that thicker stems are associated with higher photon absorption, greater excitation energy dissipation at the donor side, and enhanced energy transfer toward the PSII reaction centres. However, strongly negative correlations with TR_0_/ABS (r = −0.64), ET_0_/TR_0_ (r = −0.75), ET_0_/ABS (r = −0.75), and PI_ABS_ (r = −0.67) indicate that the efficiency of energy conversion and electron transport within photosystem II is compromised. Negative correlations with these parameters suggest that sunflower stem thickness may structurally favour growth but limit photosynthetic efficiency, a physiological trade-off in plants under stress or in conditions of intense competition for resources, which may be the case because plants were grown at different planting densities. Ghaffari et al. [49] examined the correlations between morphological and physiological traits in sunflowers under drought stress. They reported strong positive correlations between F_v_/F_m_ (TR_0_/ABS) and plant height, stem diameter, and head diameter. However, such relationships were not confirmed in the present study, where TR_0_/ABS showed significant negative correlations with head diameter (r = −0.80 during budding) and stem diameter (r = −0.64 during flowering). This discrepancy may be attributed to differences in environmental conditions, genotypic variability, or developmental stage-specific responses of the photosynthetic apparatus.

The 1000-grain weight showed a negative correlation with hectoliter mass (r = −0.63) and oil content (r = −0.85), indicating a possible competition between starch and lipid accumulation during grain development. In contrast, the study by Mijić et al. [50] reported a positive correlation between 1000-grain weight, hectoliter mass, and oil content. At the budding stage, 1000-grain weight was significantly positively correlated with parameters indicating increased absorption and initial distribution of light energy in photosystem II, such as ABS/RC (r = 0.85), DI_0_/RC (r = 0.77) and TR_0_/RC (r = 0.85). However, at the same stage, extremely negative correlations were recorded with parameters of effective electron transfer and total photosynthetic efficiency, including ET_0_/TR_0_ (r = −0.83), ET_0_/ABS (r = −0.81), RE_0_/ABS (r = −0.93), PI_ABS_ (r = −0.77) and PI_TOTAL_ (r = −0.89). Compared to the flowering stage, budding exhibits more pronounced relationships between photosynthetic parameters and storage properties, which may be important in selecting genotypes for yield and quality.

Negative correlations of hectoliter mass with parameters such as ET_0_/TR_0_ Gf (r = −0.70), ET_0_/ABS Gf (r = −0.73), and PI_ABS_ Gf (r = −0.67) indicate that higher grain density is not necessarily associated with greater photosynthetic efficiency, particularly during the grain-filling stage.

The correlation of grain yield with oil content (r = 0.57) and its extremely strong correlation with oil yield (r = 0.97) confirm the dependence of these parameters. However, Radić et al. [45] did not observe a significant relationship between grain yield and oil content in sunflower parental lines. In contrast to earlier studies on maize, which reported significant correlations between grain yield and various physiological parameters [51,52,53], this study did not find any such relationships in sunflower. This suggests that, under the specific environmental conditions and genotypic background of the present trial, physiological efficiency (e.g., TR_0_/ABS, ET_0_/ABS, PI_ABS_) is not a predictive factor for final grain yield in sunflower.

The positive correlation between oil yield and RE_0_/ABS (r = 0.53) in both budding and flowering stages indicates that plants capable of efficiently transferring absorbed energy through the entire photosynthetic electron transport chain also accumulate more oil.

The oil content exhibited a strong negative correlation with parameters per reaction centre (ABS/RC, DI_0_/RC, and TR_0_/RC), with stronger correlations observed during the budding stage than during flowering. Conversely, very strong positive correlations were detected between oil content and RE_0_/ABS in both budding and flowering stages (r = 0.83 and r = 0.76, respectively), as well as with PI_TOTAL_ in both stages (r = 0.74 and r = 0.76). Additionally, moderate positive correlations were found with TR_0_/ABS, ET_0_/TR_0_, ET_0_/ABS, RE_0_-ET_0_, PI_ABS_, and RE_0_ − ET_0_/(1 − RE_0_ − ET_0_), particularly during the flowering stage. Notably, oil content did not show statistically significant correlations with any of the measured parameters during the grain-filling stage. These findings suggest that oil accumulation is more tightly linked to photosynthetic electron transport and energy partitioning during early reproductive development.

Based on the correlation results, it was found that the relationships between morphological traits and photosynthetic parameters create complex behavioural patterns specific to sunflower genotype and developmental stage. Structural traits such as plant height, stem diameter and head size were strongly correlated with photosynthetic performance. However, correlations varied with developmental stage and planting density, indicating trade-offs between vegetative growth and photosynthetic efficiency. Photosynthetic traits at the early developmental stage (ABS/RC, TR_0_/ABS, RE_0_/ABS, PI_ABS_) were closely associated with oil accumulation, underscoring their relevance to productivity. Integral CL and Surimi CL combined morphological stability with robust photosynthetic performance, while Alexa SU and Neta SU were more sensitive. The most reliable indicators for assessing adaptability were head diameter, 1000-grain weight, ABS/RC, TR_0_/ABS, RE_0_/ABS and PI_ABS_. These findings provide practical guidelines for hybrid selection and density management to optimize sunflower yield and oil production.

## 3. Materials and Methods

### 3.1. Experimental Site

The experiment was conducted in the field of the Agricultural Institute Osijek, Croatia (45°32′ N and 18°44′ E, 90 m above sea level). The soil type in the research is a eutric cambisol with good pedophysical and chemical properties (pH KCl—6.25; humus—2–2.2%; K_2_0—37.7 mg/100 g of soil; P_2_0_5_—39.7 mg/100 g of soil).

**Figure 5:**
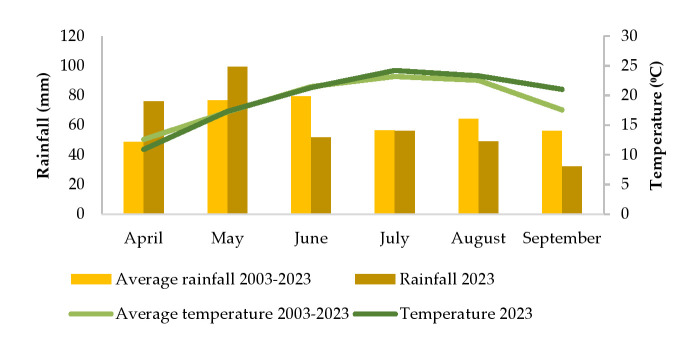
The mean monthly air temperature (°C) and total monthly rainfall (mm) during the 2023 growing season, and a long-term (2003–2023) average rainfall and temperature in Osijek–Klisa.

The mean monthly air temperature (°C) and total monthly rainfall (mm) during the 2023 growing season and a long-term (2003–2023) average rainfall and temperature in Osijek–Klisa are shown in Figure 5 (Croatian Meteorological and Hydrological Service). In April 2023, the daily air temperature was 1.7 °C less than the long-term average (2003–2023). Larger changes were observed in the increase in daily monthly temperatures in 2023 compared to the multi-year average in July (1 °C), August (0.8 °C) and September (3.4 °C). In 2023, the amount of rainfall from June to September was less than the long-term average. In June, precipitation was 35% less, in August by 24%, and in September by 43% compared to the twenty-year average. During the germination and emergence of sunflowers in 2023, about 56% more rainfall fell in April and 30% in May than the long-term average.

### 3.2. Plant Material

The experiment included five commercially relevant sunflower hybrids: 1—Surimi CL, 2—Integral CL, 3—Alexa SU, 4—Neta SU, and 5—Davero SU. These hybrids represent both Clearfield—CL (imidazolinone-tolerant) and tribenuron-methyl-tolerant (SU) technologies. Clearfield (CL) technology in sunflower production includes hybrids tolerant to the active ingredient imazamox (IMI-tolerant hybrids) and to the imidazolinone (IMI) herbicides. In contrast, ExpressSun technology includes sunflower hybrids tolerant to the active substance tribenuron (SU-tolerant hybrids) and sulfonylurea (SU) herbicides. Both technologies enable producers to achieve adequate control of a wide range of weeds after the sunflower crop emerges.

Hybrids differ in maturity group, stress and disease resistance. Surimi CL and Integral CL are characterized by high yield potential, strong tolerance to lodging and breakage, and good adaptability under drought or heat stress, with Surimi CL also exhibiting a high level of self-fertility. Alexa SU, Neta SU, and Davero SU show good tolerance to drought, key sunflower pathogens, and broomrape. Integral CL belongs to the early maturity group, Surimi CL, Alexa SU, and Davero SU belong to the mid-early maturity group, while Neta SU is slightly later. The inclusion of these hybrids allowed the assessment of genotype-specific responses to planting density under diverse environmental conditions.

### 3.3. Experimental Design

The forecrop was spring barley. The experimental design was a randomized complete block design (RCBD) with three plant densities (treatments): 17 cm plant spacing inner a row corresponding to 84,034 plants/ha (T1), 21 cm plant spacing inner a row corresponding to 68,027 plants/ha (T2), and 25 cm plant spacing inner a row corresponding to 57,143 plants/ha (T3). The intermediate spacing (21 cm; T2) represented the control treatment, as it reflects the standard sowing density commonly used in sunflower cultivation under production conditions. The denser (17 cm; T1) and wider (25 cm; T3) spacings were selected to examine the phenotypic and physiological responses of sunflower hybrids under conditions simulating potential agroecological variations associated with climate change.

The experiment was set up in three replicates. In optimal agrotechnical terms, sowing was carried out manually (using hand planters) on 14 April 2023. The sowing depth was 5 cm. The basic plot consisted of four rows, each 4 m long. The two inner rows were used for measuring properties. The row spacing between rows was 70 cm. Fertilization was carried out based on a chemical analysis of the soil with the addition of 87 kg/ha of N. After sowing and before emergence, herbicides (metolachlor + fluchloridone: 1 + 1.8 l ha) were applied, and fungicides were applied in the budding stage (boskalid + dimoxystrobin: 0.5 l ha). The harvest was conducted on 13 September 2023, utilizing a Wintersteiger small plot harvester. All agrotechnical measures applied in the wide production of sunflowers in Croatia were applied in the experiment.

### 3.4. Endpoints

#### 3.4.1. Morphological Parameters

In the field, agronomic traits: plant height, head diameter, stem diameter (measured before harvest), and number of leaves (recorded after flowering), were assessed on three plants per hybrid, treatment, and replication (i.e., three measurements per hybrid, across three treatments and three replications; *n* = 135). Plant height was measured in centimetres (cm) using a measuring tape, from the base of the plant to the top of the head. Head diameter was also measured in centimetres using a measuring tape. The stem diameter (cm) was measured just above the cotyledons using an electronic digital caliper. The number of leaves per plant included all leaves on the stem. After harvesting the hybrids, the agronomic properties of 1000-grain weight, hectoliter mass, grain yield (calculated at a moisture content of 9%), oil content (at dry matter), and oil yield were determined. All collected plants from the middle two rows made up one sample per hybrid, treatment, and replication (*n* = 45).

#### 3.4.2. Physiological Parameters

Chlorophyll *a* fluorescence (ChlF) parameters were analyzed using the JIP-test, a widely applied method formulated by Strasser et al. [21]. The Plant Efficiency Analyser (Handy PEA, Hansatech, UK) was used to record the ChlF in the morning (between 7:30 and 9:00). Before measurement, leaves were fully dark-adapted for 30 min using a leaf clip shutter plate [41]. The ChlF was induced with a saturated red-light pulse (3200 μmol m^−2^ s^−1^, peak at 650 nm). After measurements, the JIP-test parameters were processed and selected for study.

The JIP-test parameters (Table S1) used in this study are as follows: ABS/RC—absorption flux active per reactive centre (RC), DI_0_/RC—dissipated energy flux per active RC, TR_0_/RC—trapping flux per active RC, ET_0_/RC—electron transport flux per active RC, RE_0_/RC—electron flux reducing end electron acceptors at the photosystem I (PSI) acceptor side per RC, TR_0_/ABS—maximal photochemical quantum yield, ET_0_/TR_0_—the probability that a trapped exciton moves an electron into the electron transport chain beyond Q_A_^–^, ET_0_/ABS—quantum yield for electron transport, RE_0_/ET_0_—the probability that an electron is transported from reduced PQ to the electron acceptor side of PSI, RE_0_/ABS—quantum yield of electron transport from Q_A_^−^ to the PSI end electron acceptors, PI_ABS_—performance index on absorption basis, (RE_0_-ET_0_)/[1 − (RE_0_-ET_0_)]—electron transport from PQH_2_ to final PSI acceptors and PI_TOTAL_—performance index for energy conservation from exciton to the reduction in PSI terminal acceptors.

Physiological properties of photosynthetic efficiency were determined on three developmental stages of sunflower: budding, flowering, and grain-filling. These stages were selected because they represent critical periods during which plants are most sensitive to environmental conditions and exhibit significant variations in growth and photosynthetic activity. Measurements were performed on the second full development leaf (according to Schneiter et al. [54], whether each leaf is fully developed if it is 4 cm), counting from the top of the plant downward, of each hybrid, with three leaves assessed per hybrid, treatment, and replication (three measurements per hybrid, per three treatments and three repetitions; *n* = 135).

### 3.5. Data Analyses

Data obtained from measurements of the investigated properties were systematized by hybrids and treatments and processed using analysis of variance (ANOVA) and Fisher’s least significant difference (LSD) test (*p* = 0.05). Comparisons were made only within each hybrid between sowing density treatments to evaluate the effects of plant density on morphological and physiological traits. Morphological and physiological parameters were compared using Pearson’s pairwise correlation test. The statistical software package used for analyses was Statistica software (TIBCO Software Inc., version 14, Palo Alto, CA, USA).

## 4. Conclusions

Based on the analyzed data, this study demonstrates that sunflower hybrids exhibit significant variability in agronomic performance and photosynthetic responses depending on planting density and developmental stage. Higher planting densities generally promoted higher plants but had a negative impact on head and stem diameters, 1000-grain weight, and hectoliter mass. Chlorophyll *a* fluorescence parameters varied most notably during the budding stage, indicating that early reproductive development is critical for PSII activity and energy efficiency.

Among the tested hybrids, Alexa SU and Neta SU exhibited the greatest physiological sensitivity to planting density, reflecting their high plasticity and potential adaptability under lower densities. In contrast, Surimi CL and Integral CL showed more stable agronomic and physiological performance across densities, suggesting their suitability for denser planting systems. Davero SU displayed moderate adaptability, with no substantial decline in yield or physiological efficiency at intermediate densities. The observed hybrid-specific responses to density likely arise from genotypic differences in plant architecture, leaf anatomy, and photosynthesis-related regulation.

Correlation analysis highlighted a developmentally dependent and often antagonistic relationship between morphological traits and photosynthetic efficiency. These findings suggest that optimizing plant density and selecting appropriate genotypes requires a careful balance between structural biomass development and physiological function, particularly during early reproductive stages.

Importantly, this study represents a preliminary investigation within an ongoing project to assess the impact of planting density on sunflower hybrid performance from both agronomic and physiological perspectives. These findings provide forward-looking insights into sunflower breeding, hybrid selection, and density management under variable environmental conditions, offering practical guidance to enhance yield stability and physiological resilience. Continued multi-year experiments will further validate these initial results and help develop hybrid-specific agronomic recommendations.

## Figures and Tables

**Table 1:** ANOVA for the effect of plant density (treatment) and sunflower hybrid on the investigated agronomic properties.

Factor	Number of Leaves	Plant Height	Head Diameter	Stem Diameter	1000-Grain Weight	Hectolitre Mass	Grain Yield	Oil Content	Oil Yield
Treatment (T)	ns	*	*	*	*	ns	*	*	*
Hybrid (H)	*	*	ns	*	*	*	*	*	*
T × H	ns	ns	ns	ns	ns	ns	*	*	*

ns—not significant, *—significant.

**Table 2:** ANOVA for the effect of plant density (treatment) and sunflower hybrid on the investigated physiological properties.

Factor *	ABS/ RC	DI_0_/ RC	TR_0_/ RC	ET_0_/ RC	RE_0_/ RC	TR_0_/ ABS	ET_0_/ TR_0_	ET_0_/ ABS	RE_0_/ ET_0_	RE_0_/ ABS	PI_ABS_	(RE_0_ − ET_0_)/ [1 − (RE_0_ − ET_0_)]	PI_TOTAL_
Stage (S)	*	*	*	ns	*	*	*	*	*	*	*	*	*
Treatment (T)	*	*	*	ns	ns	*	*	*	ns	*	*	ns	*
Hybrid (H)	*	*	*	*	*	*	*	*	*	*	*	*	*
S × T	*	*	*	*	ns	ns	*	*	ns	*	*	ns	ns
S × H	*	*	*	*	*	*	*	*	*	*	*	*	*
T × H	*	*	*	ns	ns	*	*	*	*	*	*	*	*
S × T × H	*	*	*	*	*	*	*	*	*	*	*	*	*

The parameters are described in Section 3.4.2; ns—not significant, *—significant.

## Data Availability

The data presented in this study are available on request from the corresponding author.

## Supplementary Materials

The following supporting information can be downloaded at: https://www.mdpi.com/article/10.3390/plants14223446/s1, Table S1: JIP-test parameters with associated formulas, Table S2: Correlation coefficients between morphological and physiological properties, Table S3: Main agronomic and physiological traits of the investigated sunflower hybrids.
